# The Dynamics of Democracy, Development and Cultural Values

**DOI:** 10.1371/journal.pone.0097856

**Published:** 2014-06-06

**Authors:** Viktoria Spaiser, Shyam Ranganathan, Richard P. Mann, David J. T. Sumpter

**Affiliations:** 1 Institute for Futures Studies, Stockholm, Sweden; 2 Department of Mathematics, Uppsala University, Uppsala, Sweden; National Scientific and Technical Research Council (CONICET)., Argentina

## Abstract

Over the past decades many countries have experienced rapid changes in their economies, their democratic institutions and the values of their citizens. Comprehensive data measuring these changes across very different countries has recently become openly available. Between country similarities suggest common underlying dynamics in how countries develop in terms of economy, democracy and cultural values. We apply a novel Bayesian dynamical systems approach to identify the model which best captures the complex, mainly non-linear dynamics that underlie these changes. We show that the level of Human Development Index (HDI) in a country drives first democracy and then higher emancipation of citizens. This change occurs once the countries pass a certain threshold in HDI. The data also suggests that there is a limit to the growth of wealth, set by higher emancipation. Having reached a high level of democracy and emancipation, societies tend towards equilibrium that does not support further economic growth. Our findings give strong empirical evidence against a popular political science theory, known as the Human Development Sequence. Contrary to this theory, we find that implementation of human-rights and democratisation precede increases in emancipative values.

## Introduction

Despite cultural, political and economic differences across different countries, more and more countries are moving toward more democratic regimes, with higher levels of civil liberties [1], [2]. At the same time rising levels of education and standards of living in many parts of the world have given more and more people higher existential security [3]. Parallel to these phenomena, the World Values Survey has found that cultures and therefore cultural values are changing too [4].

What is the relation between these various phenomena that have transformed various societies? In the 1950s, Lipset [5] provided empirical evidence for a positive relation between socioeconomic development and political democratisation. Later studies mostly confirmed this positive correlation [6]–[10]. Recently, we re-explored the relation between democratisation and economic development, accounting for possible non-linearities [11]. We found that democracy does not not grow linearly with GDP per capita, instead we see rapid and sudden democratisation once GDP per capita has surpassed a certain threshold. If GDP per capita is below this threshold, economic growth will not cause democratisation. On the contrary, countries starting with a certain level of democracy experience democratic decline.

While our earlier work identified a threshold effect, it does not reveal the roots of the relation between democracy and economic development. And in fact, GDP is often considered as inappropriate to measure the overall economic development level of a country [12]–[15]. Moreover, democracy and GDP are just two of a large number of factors – including education health and cultural values – involved in the process of development. It is known that together these and other socio-economic factors provide favourable conditions for democratisation [5]–[7], but it remains unclear what the relation between them and democratic change precisely is [5], [8], [16]. There exists also an alternative measure for nations’ socio-economic development, HDI (Human Development Index). The HDI is a combined metric of education, wealth and life expectancy [17], [18] and therefore gives a fuller picture of development than GDP per capita alone. Our aim in this paper is to re-examine the black box relationship between democracy, socio-economic development and cultural change [5]–[10], [19].

It is possible now to attain this objective because of the recent availability of cross-national time-series data on indicators measuring socio-economic, political and cultural development. Table 1 lists a set of ten socio-economic, democratic and cultural value indicators that we use in this paper. These indicators are available for many of the world’s countries over the last 30 years. For socio-economic progress we primarily use HDI and respective indicators included in HDI [17], [18]. We also use measure of both human-rights and effective democracy [20]–[22] and cultural changes measured in the World Values Survey [20], [23]. Using such a varied selection of longitudinal data will allows us to identify interactions between the indicators which capture the overall pattern of development. The data set is described in full in the next section.

**Table 1:** Indicators used in the analysis.

Indicators	Range	Components	Source	Years	Countries		
**Socio-economic indicators**							
log GDP per capita (G)	5 to 12	-	World-Bank (http://data.worldbank.org)	1800–2009		213	
Humand Development Index HDI (H)	0 to 1	UN education index, life expectancy, GNI per capita	UNDP (http://data.un.org)	1980–2012		193	
log GNI per capita (G)	5 to 12	-	World-Bank (http://data.worldbank.org)	1980–2012		213	
UN education index (I)	0 to 1	mean years of schooling, expected years of schooling	UNDP (http://data.un.org)	1980–2012		193	
Life expectancy (L)	42 to 83	-	World-Bank (http://data.worldbank.org)	1960–2008		213	
Female education (F)	0 to 13	-	Barro-Lee Educational Attainment Dataset (http://barrolee.com) [63]	1960–2004		146	
**Democracy indicators**							
Human-rights democracy (D)	0 to 1	political rights score, civil liberties score, human-rights performance score	Freedom House [64], [65], Cingranelli & Richards Human Rights Data Project (CIRI) [66], [67]	1980–2006		187	
Effective democracy (D)	0 to 100	political rights score, civil liberties score, corruption score	Freedom House [64], [65], World Bank (Worldwide Governance Indicators (WGI) data) [68]	1996–2006		150	
**Cultural Values indicators**							
Emancipative values (E)	0 to 1	-	World Value Survey (http://www.wvsevsdb.com)	1981–2011		65	
Self-expressive values (S)	−2 to 2	-	World Value Survey (http://www.wvsevsdb.com)	1981–2011		65	

We are not the first researchers to attempt to identify between-country similarities in human development. Indeed, the approach dates back to modernization theory of the 1950s and 1960s [5], [24]–[26]. Currently, one prominent modernization theory is the Human Development Sequence. It proposes that cultural values mediate the effect that economic development has on democratisation [20], [27]–[29]. The sequence describes a linear relation where economic progress enables change of cultural values which ultimately leads to democratisation. Economic development provides the opportunities and means for a self-expressive and emancipated life and the desire to shape one’s own life provides a motivation to change the rules by which people are governed, therefore demanding more democracy [20], [28]–[30]. This theory has been disputed [19], [31]–[33], and it remains an open question to what extent it can explain commonalities in the development of very different countries.

More recently, Abdollahian et. al. [34] took a more systematic approach to the available data. They wrote down differential equations for changes in GDP, self-expressive values and democracy, based on the assumptions of the Human Development Sequence. They show that the model parameters fit the available time series data. However, these authors do not test alternative models. It is always possible to find the parameter set for a given model that best fits the data, but the important question is whether there are other plausible models that perform even better. Here we properly account for this limitation. We do not start with a strict hypothesis about the underlying patterns in human development, but rather identify the best models that fit the available data. We adopt the novel Bayesian dynamical systems approach detailed in Ranganathan et al. [11], but in the current paper account for all relevant and available indicators of human development.

We proceed as follows. We first describe the “Data” we use in more detail. Then in the “Results” we present the best fit models of the complex dynamic interactions between the Human Development Index (HDI), cultural values and human-rights democracy. In section, “Role of the HDI components” we look at the role of the components of HDI in relation to democracy and cultural values. In section “Testing the Human Development Sequence”, we test the Human Development Sequence theory assumptions and show that it is not particularly well supported by the available data. We finally discuss our overall results and suggest an alternative model of human development based on our analysis. In the “Material and Methods” section at the end we describe in more detail the methodological approach we used in our analysis. A comprehensive supplementary material includes further details of the data and additional results used to support the main conclusions.

### Data

We used ten different indicators in our analysis: six socio-economic indicators, two democracy indicators and two cultural values indicators (see Table 1). The main socioeconomic indicator, HDI (Human Development Index), is a composite index that combines measures for education, life expectancy and Gross National Income (GNI) per capita [17], [18]. Additional analyses were carried out on Gross Domestic Product (GDP) per capita and female education. GDP per capita was used to test the Human Development Sequence. Female education (mean years of girls’ schooling) was included in the analysis because it is an indicator that education is equally open and accessible to all parts of the population. Moreover female education measures gender equality.

To operationalise democracy we used two measures: 1) The human-rights weighted democracy index consists of civil liberties, political and human rights indices measured in the Freedom House and Cingranelli & Richards Human Rights data project [20]. It is our main democracy measure and and we will refer to it as human-rights democracy. 2) The effective democracy index is also based on Freedom House civil liberties and political rights indices, but weighted by a rule of law index [21], [22]. We used it to test our results against earlier fitting of the HDS theory.

We measured cultural values with 1) the World Values Survey emancipative values index, which is our main cultural values index and with 2) self-expressive values [20], [23]. Emancipative values measure “decision-making freedom of the individual human being and the equality of all human beings in this decision-making freedom” [35]. Self-expressive values are to a great extent equivalent to emancipative values, but encompass a larger variety of different measures. For instance, the self-expressive index includes prioritisation of environment protection, quality of life, accomplishment and economic liberties, as well as measures of social trust and respondent’s life satisfaction [23]. We use both values in order to test result robustness and to reproduce earlier tests of the HDS theory.

All indicator values, available from 65 countries for the time period of 1981 to 2006, were scaled to provide indexes between 0 and 1 for better parametrisation (see Table 1). For more details of the indicators used see Supporting Information section S12 in File S1.

## Results

In order to identify interactions between indicators without an *a priori* picture of how these interactions should look, we adopt the Bayesian dynamical systems approach described in Ranganathan et al. [11]. We fit differential equations for the rate of change of each indicator as a function of the level of the indicator itself and the level(s) of other indicator(s) in the previous year. The function is polynomial, consisting of polynomial terms that cover diverse linearities and nonlinearities. These multiple linear and non-linear terms and their combination give a large number of alternative models. We calculate the Bayes factor to fairly compare models of different complexity. Since we are considering ten indicators we build our analysis up stepwise.

### Democracy, HDI and Emancipative Values

**Figure 1:**
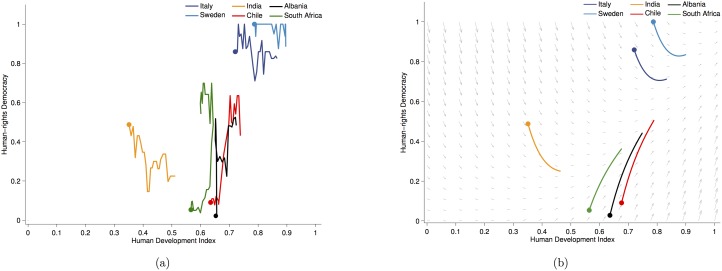
Phase portrait of changes in human-rights democracy values (x-axis) against changes in HDI (y-axis). The coloured lines give temporal changes in representative countries, starting from the solid dot for 1981. In (a) data is used to draw the trajectories. In (b) best-fit models are used to predict the changes given initial values in 1981. The arrows represent a vector field showing changes according to the best fit models for human-rights democracy (equation 1) and HDI (equation 3).

The starting point is the relationship between HDI and human-rights democracy. Figure 1(a) shows trajectories for six example countries for the period 1981–2006. The figure suggests both, interesting between country differences and some similarities in the change of the trajectories over time. For example, despite fluctuations there was an overall decline in democracy in India between 1981 and 2006, while India’s HDI was increasing at the same time. For South Africa, Albania and Chile we see a common dynamic of rapid and radical democratisation irrespective of the fluctuations preceding and following the democratisation wave. Finally, Sweden and Italy also have similar trajectories: a minor democratic decline followed by a recovery but an overall high democratic level. All countries, with the exception of South Africa, increased their HDI between 1981 and 2006.

To capture the general trend we used the entire dataset of 65 countries to calculate the best of all possible two variable models relating current levels of human-rights democracy (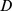) and HDI (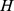) to the rate change in human-right democracy (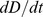). The best fit model model, i.e. that with the largest Bayes factor (see “Materials and Methods” for details on Bayes factor), is
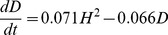
(1)


This model indicates that human-rights democracy increases non-linearly with HDI, but is self-limiting as democracy is not likely to grow once it reached a high level. Rather than simply increasing as a function of HDI, human-rights democracy increases only when 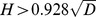. This relationship captures differences in how different countries have changed over the last 30 years, and is represented in the phase portrait in Figure 1.

The non-linear relationship between economic development and democracy accounts for the difference in the development trajectories of different countries. Human-rights democracy does not always increase with growing HDI. Rather, a country must first reach a threshold economic development before democratisation starts. If HDI is below that threshold, democracy will regress, even if HDI itself is growing, as is seen for India. The non-linear relation also captures the phenomena of sudden democratic changes [36]. The level of democracy can remain low for a long time, with some fluctuations but no real progress towards greater democracy. However, once the threshold HDI is reached the democratic changes occur rapidly and radically. This is exemplified by Chile, South Africa and Albania, all of which experienced rapid democratisation in the late 1980s and early 1990s.

**Figure 2:**
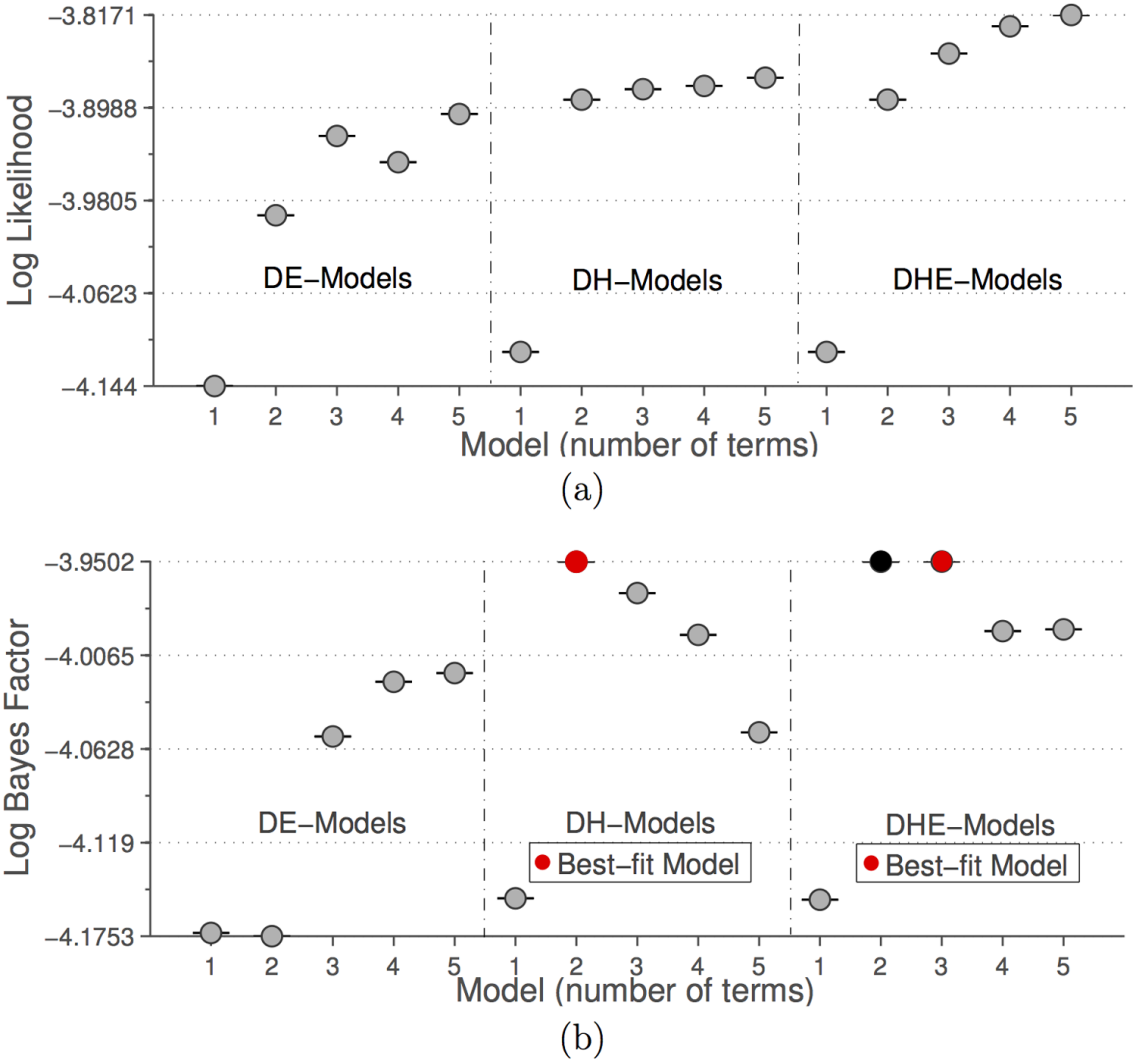
Log Likelihood and Log Bayes factor for models of change in democracy as a function of the variables and the number of terms allowed in the model: 
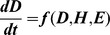
. Number of terms refers to the terms in the polynomial 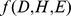. See “Material and Methods” section for details on the fitting method. DE-models are of a form 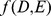, i.e. terms containing human-rights democracy and emancipative values as predictors, DH-models use human-rights democracy and HDI as predictors in 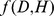 and DHE-Models models use all three variables as predictors. The two best-fit models (marked in red) refer to equations 1 and 2. The two-term DHE-model (marked in black) is identical to the best-fit two-term DH-model, equation 1.



We now turn our attention to a possible effect of cultural values on democracy. The Human Development Sequence proposes that democracy should increase as a function of emancipative values (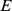) [27], [28]. This assumption is supported by the data. Figure 2 shows both Log Likelihood and Bayes factor for all alternative best-fit models of varying complexity (number of included predictors and number of included polynomial terms) for 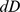 as a function of 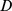 itself, 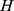 and 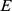. From this we can see that models combining human-rights democracy, HDI and emancipative values do not better predict changes in democracy than equation 1 with only 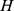 and 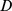 as predictors. From a large number of all possible models including all three predictors, the overall best-fit model, i.e.

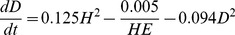
(2)does have a Bayes factor roughly equal to that of equation 1. The additional 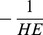 term means that in two countries with identical HDI, the country with lower emancipation will develop democracy more slowly. The evidence for including the extra term is however marginal, and HDI remains a much more important predictor of democratic change than emancipative values. These results were also robust to replacing emancipative values with self-expression values (see section S1 and Figure S1 in File S1).

Model 1 implies that HDI has a non-linear effect on changes in democracy, but does democracy have a positive effect on changes in HDI? Factors predicting increases in HDI are notoriously difficult to identify [10], [37], [38]. We also find that changes in HDI are independent of levels of both human-rights democracy and emancipative values (see section S2 and Figure S2 in File S1). The highest Bayes factor model implies a constant rate of growth of HDI with neither democracy nor emancipative values having a significant effect on changes in HDI, i.e.
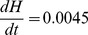
(3)


Our analysis thus far suggests that cultural change is not a decisive predictor of democratic change or socio-economic development. But how is cultural change affected by political and socio-economic change? We found that emancipation increases as a function of both economic development and human-rights democracy. Specifically, emancipative values increase in proportion to their product, and the model.
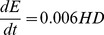
(4)has the highest Bayes factor (see section S3 and Figure S3 in File S1). Emancipative values are adopted most rapidly by major parts of the population when both economic development and human-rights democracy are well established.

The system of equations 1, 3 and 4 provide a description of the most probable dynamic of development across countries for the last 30 years. Equation 3 shows that HDI appears to evolve independently. Equations 1 and 4 show that HDI then drives both the emergence of human-rights democracy and of emancipative values. Human-rights democracy also contributes to an increasing emancipation (equation 4). These results are robust, in the sense that when we look at the highest Bayes factor models with one more or one less term than in equations 1 to 4 they do not radically change the interpretation of the model. For example, including a third emancipative values term in equation 1 reveals a rather weak effect from emancipative values on human-rights democracy.

Repeating the above analyses with the effective democracy measure, rather than human-rights democracy, we see that the former measure does not have the same effect on emancipative values. The best predictor of changes in 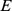 in this case is HDI alone. This would suggest that the crucial component of democracy for emancipative values to spread in a population appears to be human rights. Otherwise, the results for changes in HDI and changes in effective democracy are largely consistent with those we found using human-rights democracy index (see section S4 and Figure S4 in File S1).

### Role of the HDI Components

We have identified relationships between growth of human-rights democracy and levels of HDI, but HDI itself is a complex measure and encompasses various phenomena. To open up the HDI black box, we looked at how changes in human-rights democracy are predicted by its components: life expectancy (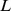), UN education index (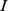) and log GNI per capita (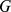).

We found that education and GNI are better predictors for changes in human-rights democracy than life expectancy (see section S5 and Figure S5 in File S1). The model which combines positive effects of education and GNI per capita on human-rights democracy has the highest Bayes factor for predicting human-rights democratic change:
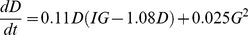
(5)


Education is often identified as a mediator in the positive relation between socio-economic development and democracy [7], [9], [39], [40]. In this model education interacts with GNI per capita, an indicator for the standard of living of people of a given country and it is this product that contributes to democratisation.

The lack of predictability of HDI in equation 3 is also somewhat unsatisfactory. It is surprising that democratisation and cultural values appear to have no impact on socio-economic development. It is often argued that socio-economic development actually benefits from democratic conditions [41], [42]. Breaking HDI in to its components explains why we obtained equation 3: emancipative values have opposite effects on the different components. Specifically, life expectancy is predicted to increase in societies with high levels of emancipative values, with the model
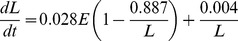
(6)providing the highest Bayes factor. On the other hand, growth of GNI per capita is slowed down by emancipative values, and the most probable model is



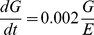
(7)This negative effect of emancipative values on GNI per capita neutralises the positive effect on life expectancy, and thus explains why no overall effect is measurable from emancipative values on HDI (see section S6 and Figure S6 in File S1).

Finally, education, the third HDI component, is best predicted by a constant growth rate (see section S6 and Figure S6 in File S1) with neither emancipative values nor democracy being decisive for predicting growing levels of education in a population, i.e.
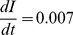
(8)


However, if we replace the education measure with an index for female education, emancipative values do predict increases in female education, 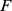, and the model
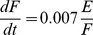
(9)has the highest Bayes factor of those models involving 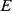 and 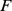 (see section S7 and Figure S7 in File S1). Female education may be seen as a measure of equal access to education and gender equality. In itself, female education is as good predictor for human-rights democracy as the overall education index. The model with the highest Bayes factor, i.e.
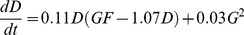
(10)combines a positive effect of female education and GNI per capita on democracy. Comparing this model with equation 5 suggests that female education and general education are equivalent with respect to their effect on human-rights democracy (see section S8 and Figure S8 in File S1).

In the previous section, we showed that HDI and human-rights democracy both predict increases in emancipative values (equation 4). The question now is which HDI components are most important for emancipative values to spread in a population? We looked at the role of the three components of HDI in predicting changes in emancipative values (see section S9 and Figure S9 in File S1 and see section S10 and Figure S10 in File S1 for female education effects) and found that life expectancy and human-rights democracy constitute the best predictors for rising emancipative values, i.e.
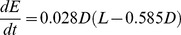
(11)has the highest Bayes factor (see section S11 and Figure S11 in File S1). This would suggest that life-expectancy provides a more natural measure of overcoming existential concerns – a precondition for emancipation according to the HDS theory – than, for example, GNI.

### Testing the Human Development Sequence

Our analysis so far has already challenged several assumptions of the Human Development Sequence (HDS) theory. To quantify the difference in fit between our model and HDS theory we compare the fit of our models to the fit of differential equations models proposed by Abdollahian et al. [34]. These authors used GDP per capita (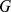), self-expressive values (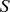) and democracy (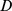) [34] as indicators. They proposed the following models:
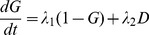
(12)

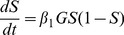
(13)

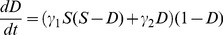
(14)


Estimating the model parameters using a least-squares methods they concluded that overall the models fit the data quite well. They found that economic growth was poorly predicted by democracy (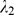 was not significantly different from zero). Therefore, they revised their original model and removed 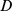 as predictor. They also found that 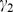 showed a negative sign, indicating that democracy is not self-reinforcing. But they did find evidence for 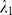, 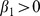 and 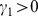 as predicted by HDS.

**Figure 3:**
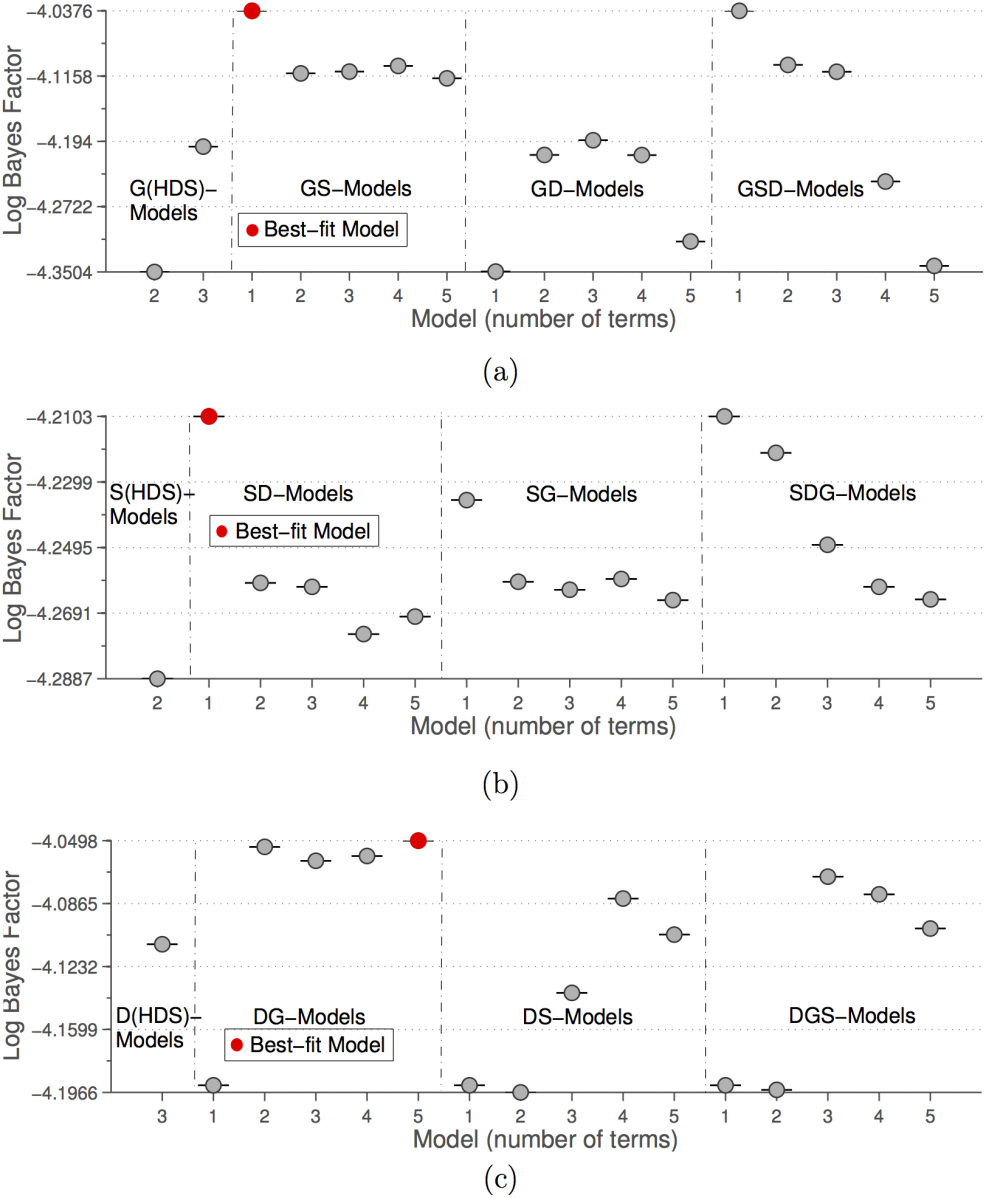
Comparing HDS and data-derived models for GDP per capita (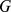), human-rights democracy (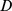) and self-expressive values (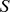). (a) G(HDS)-Models are the two models suggested by Abdollahian et al. (2012) [34] to model changes in GDP per capita (equations 12 and the same equation without 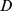 as predictor) based on HDS theory. GD-, GS- and GSD-Models are models for changes in 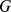 with either 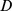, 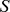 or both predictors, derived from the data. The Best-fit Model refers to equation 15. (b) S(HDS)-Model is the model suggested by Abdollahian et al. (2012) [34] to model changes in self-expressive values (equation 13) as predicted by the HDS theory. SD-, SG, and SDG-Models are models for changes in 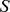 with either 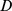, 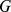 or both predictors, derived from the data. The Best-fit Model represents equation 16. (c) D(HDS)-Model is the model suggested by Abdollahian et al. (2012) [34] to model changes in democracy according to the HDS theory (equation 14). DG-, DS- and DGS-Models are models for changes in 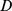 with either 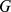, 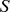 or both predictors, derived from the data. And the Best-fit Model here is shown in equation 17.



The above models (equations 12 to 14) are just one subset of the models tested in our analysis, where we look at all polynomial combinations of 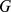, 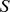 and 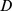 and others as predictors of change. Figure 3 shows the Bayes factors for models suggested by [34] in comparison to the best fit models when allowing for all polynomial terms. With our approach, the models with the highest Bayes factor are

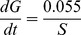
(15)

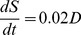
(16)

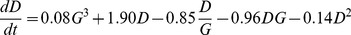
(17)


These models outperform HDS-based models to a very significant degree, with not only the best model having a higher Bayes factor, but a whole range of other models outperforming equations 12 to 14. In particular, the equation 13 for changes in self-expression is very poorly captured by HDS theory in comparison to a simple linear growth relation to democracy.

The equations for GDP and democracy also differ substantially from the predictions of HDS. Equation 15 confirms our earlier result that economic growth is slowed with emerging self-expressive values. Human-rights democracy itself is predicted in equation 17 to change as a function of GDP per capita, rather than by self-expressive values. Equation 17 is rather complicated, and it is worth noting that the second best model is the simpler relation
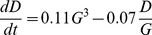
(18)which is again similar to our earlier findings for HDI (equation 1). Both equations 17 and 18 show that GDP per capita has a positive but non-linear effect on democracy, while democracy’s growth is mostly limited by high levels of democracy itself. Although having slightly different functional forms, these equations produce similar relationships between socio-economics, cultural values and democracy as we saw in previous sections. The results in the previous section were not dependent on the particular variables chosen, but are robust to changes in the measurements used.

## Discussion

**Figure 4:**
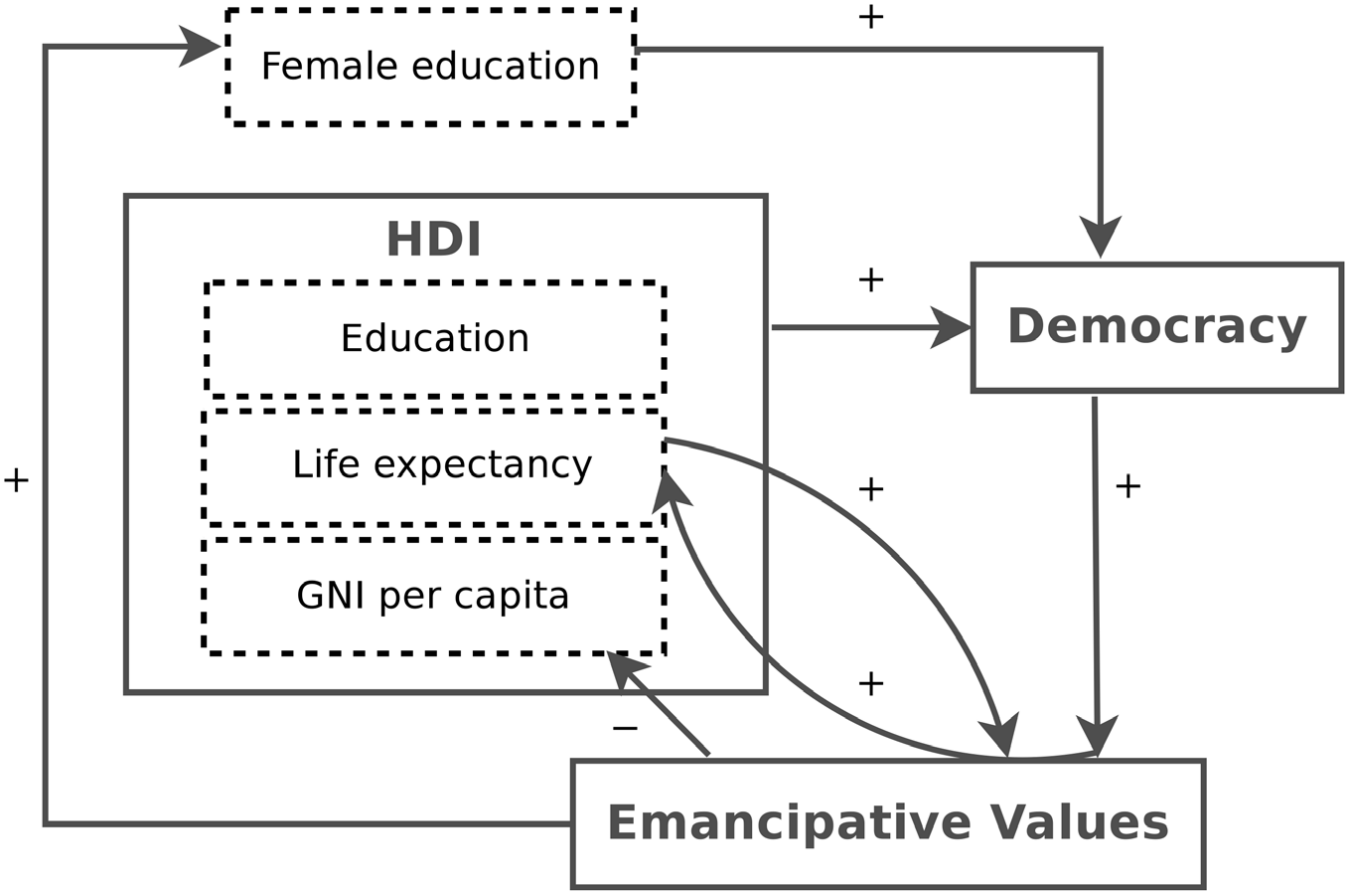
Human development model based on the equations (see **Table 2**) found in our analysis. The feedbacks positive (+) or negative (−) indicate the sign of terms in the models selected on the basis of the Bayes factor.

Given the complexity of interactions involved in human development, we cannot expect one mathematical model to account for all the variability in this process. We have however identified a small set of equations that fit the changes in economic, social, political and value indicators over the past 30 years (see Table 2). The implications of these equations are summarized in Figure 4. They do not correspond to a linear sequence, but instead a set of dynamic thresholds, feedbacks, accelerating and decelerating as well as limiting effects. We now discuss in more detail the important steps in this cycle.

**Table 2:** Set of equations describing relations in Figure 4.

Equation	Relation
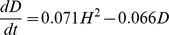	HDI 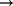 Democracy
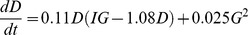	
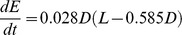	Democracy 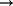 Emancipative Values
	Life expectancy 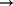 Emancipative Values
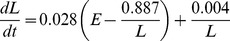	Emancipative Values 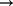 Life expectancy
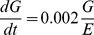	Emancipative Values 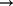 GNI per capita
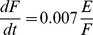	Emancipative Values 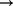 Female education
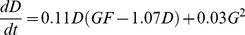	Female education 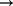 Democracy

Starting with democracy, we find that improved education and a certain standard of living as expressed in GNI per capita lead to an improved political climate (equation 5). One micro-level explanation for this relationship is that education makes people more critical and autonomous and educated people are likely to demand political changes once they have reached a certain standard of living and financial security that relieves them from daily existential worries and allows for long-term planning of society. This hypothesis is supported by Mani et al. [43], who found in lab and field experiments that cognitive performance is reduced in individuals experiencing severe financial concerns. This effect prevails when controlling for people’s intelligence, education and various other factors. Severe financial concerns made it difficult for people to think clearly and to plan the long term. Both our macro-level patterns and their micro-level experiments suggest that poverty itself inhibits democratisation, without the need for a mediating factor.

For cultural values, we found that emancipative values increase with existential security, with life expectancy playing the most important role. Equation 11 and our robustness tests with effective democracy index suggest that existential security seems to go beyond life expectancy, and include security granted by human rights. On the other hand, and contrary to earlier suggestions [20], [23], GNI or GDP per capita did not directly lead to an increase in emancipation. Economic development alone is not sufficient to free people from existential concerns and some level of human rights and therefore of democratisation is also required.

Socio-economic development is affected by cultural values (equation 7). Earlier studies on cultural values’ effect on economic growth have come to different, sometimes contradictory conclusions [44]–[47]. We find that emancipative values are associated with a slowing of economic growth, possibly as a result of a change of emphasis from materialistic to post-materialistic goals. On the other hand, countries with higher emancipative values exhibit increases in life expectancy and female education (equations 6 and 9 respectively). As such, while emancipative values may foster equal access to education, gender equality and a healthy life they do not lead to further accumulation of wealth.

In summary, we show that a critical level of Human Development Index triggers democratisation and then the emancipation of the population. We find that, human-rights based democratisation precedes increases in emancipative values, rather than the other way around. Moreover, once countries reach high levels of democracy and emancipation, they tend towards equilibrium in terms of economic growth. Higher emancipation appears to limit further growth of wealth.

The results we have presented are the best fit models to the available data. We assumed no *a-priori* pattern in human development, and by using a Bayesian approach identified genuine statistically significant relationships within the data. There are several reasons to believe that the relationships we have identified are robust. Firstly, the use of Bayes factor ensures that we do not specify overly complex models. Secondly, by looking at alternative indicators and including different numbers of terms we ensure that the results are not sensitive to measurement changes. Finally, by including a full range of non-linear terms, we capture the complex interactions inherent in the data.

Our approach contrasts strongly with that taken in specifying the Human Development Sequence (HDS), where the overall hypothesis was first postulated and then ‘tested’ against data. This testing involves comparing the predictions of the theory against a null hypothesis in which there is no detectable pattern in the data [20]. Such an approach fails to account for the vast space of possible models about human development. The fact that one model provides some predictive power hides the fact that there may exist multiple other theories and models that provide just as good or even better predictions. Our approach simultaneously tests all models and reveals those which fit the data most robustly. We find that some predictions from the Human Development Sequence theory are supported, but other central predictions are not. The most notable inconsistency between data and HDS theory is seen in emancipative values, which only poorly predict democratic change. On the other hand, the assumption that emancipative values increase with existential security is justified, but should be measured in terms of life expectancy and human rights, and not GNI or GDP per capita.

One of the problems with describing and predicting the evolution of human social systems is that many written alternative explanations of social change appear to ’make sense’ in terms of our own experience and the rational motives of agents [20]. One could argue that we have simply provided one more sensible story of this type. However, the story we provide here accounts for the inherent non-linearities in human development and is shown to be the best fit to the best available macro-level data. Moreover, the directional links in the cycle shown in Figure 4 are consistent with micro-level analyses of the World Value Survey data. Emancipative values in individuals are higher for those living in the more democratic countries [20], those with the higher household incomes [20] and those with the higher formal education [20]. Individuals with higher formal education tend to support democratic institutions and democratic norms [49]–[53]. Ideally, we would like to see how these micro-level variables change within the same individual over time. For example, to determine the relative importance of income and human rights experienced by an individual on their degree of emancipation, we would need data on how they and their children changed in these factors over a 20 or 30 year period. At present this type of panel data does not exist.

The fact that we have been able to carry out the current analysis relies firstly on the new methodological approach we have developed [11]. Secondly, it is possible because of the fact a comprehensive time series data for indicators has recently become available (Table 1). As more data becomes available, for instance data on the current economic crisis or on ecological factors, the best fit model may change, and our interpretation of the data should also change. This pragmatic point is essential. It places an emphasis on exploratory methods for finding the best interpretation of the available data [54]–[56], rather than providing a justification of an already existing verbal or theoretical argument. As society becomes more data rich, these methods are going to play an increasing role in how we interpret the past, predict the future and develop theories.

## Materials and Methods

We adopted a novel data-driven mathematical modelling approach to analyse dynamic relationships in the yearly changes of above mentioned indicators. The methodological approach is explained in detail in our recent paper [11]. Our basic approach to understanding interactions between indicators is to model changes in one variable between times 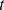 and 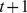 as a function of all included model variables at time 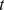. We fit ordinary differential equations to country-level data on indicators, that consist on the right-hand side of polynomial terms to capture various linear and non-linear effects. In a two variable model case we fit a model to describe the average yearly changes in 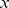 as a function of both 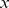 and 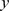,
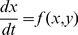
(19)


For example, consider indicator variables for HDI (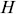) and democracy (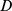). We would seek a best-fit model for changes in democracy 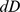 as a function of 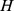 and 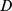 and in order to test a wide range of possible interactions, we fit models of the form:
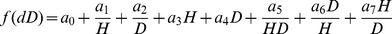


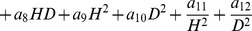
Where the values 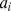 are coefficients to be fitted from the data in a multivariate regression. These terms do not provide an exhaustive set of all possibilities, but allow enough flexibility to capture possible non-linearities in the system. Still, sometimes it might be necessary to include cubic terms (e.g. in equation 6 and 7) to capture multistage dynamics for instance (see also [11]).

There are 13 possible models with one term and, in general 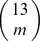 possible models with 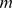 terms. We fit all 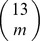 possible models with 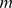 terms, restricting 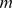 to be up to six, computing the Log Likelihood 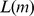 for each model using Ordinary Least Square (OLS) regression. This provides the Maximum-Likelihood estimate of the parameter values (assuming *iid* Gaussian residuals). In the first stage of our fitting process, we aim to rapidly narrow our search by finding the Maximum-Likelihood model (equivalently the model that minimises the sum of squared errors with the observed data) for each possible number of terms, 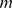.

The models preselected on the basis of maximum log likelihood are then fitted again using a Bayesian model selection approach [57]–[61]. Specifically, we calculate the Bayesian marginal likelihood, which we call “Bayes factor” 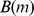, which expresses the probability of the data conditioned *only on the model*, without selection of a specific set of parameter values. Calculating the Bayes factor is the likelihood averaged over the parameter space with a prior distribution defined by 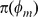.
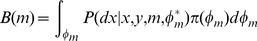
(20)


The Bayes factor compensates automatically for the increase in the dimensions of the model search space, as the prior probability on any particular choice of parameters is reduced in proportion to the number of possible parameter values [60]. We choose a non-informative prior distribution [62]. For example, 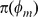 can be chosen to be uniform over a range of possible parameter values. This range of values is chosen to include all feasible values but to be small enough for the integral to be computed using Monte Carlo methods.

The key idea in our approach to modeling additional indicators is to look at how model fit improves as we add further indicators. Model complexity depends now both on the number of terms and the number of variables. For three variable models we would like to determine whether or not we require all of these variables to model their rates of change. To do this, we calculate Bayes factor for models including all three indicators and compare them to those including just pairs of indicators.

In a three variable model case we fit a model to the average yearly changes in 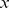 as:
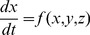
(21)


In making a fitting, we first assume that the yearly changes are polynomial functions of 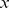, 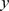 and 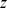, using a restricted range of polynomial terms as in the two-variable fitting. The model for change in 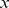 may be expressed now by any combination of the following 33 polynomial terms, for example with the three indicators HDI (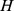), democracy (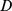) and emancipative values (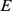):
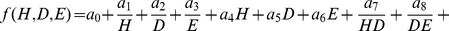


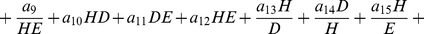


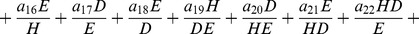


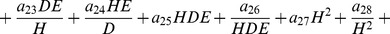


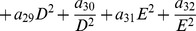



For three indicators we have now 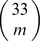 possible models with 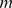 terms. We generally restrict our analysis to those with up to 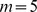 terms because of the rapidly increasing number of possible models with 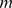. By plotting 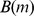 for three variable models as a function of 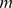 and comparing this to 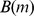 for two variable models we can assess the utility of adding a third explanatory variable to the model. Further details of methodology are given in [11].

## Supporting Information

File S1 **Supporting Information document with all Supporting Information figures and additional descriptions, explanations and analyses.**
(PDF)
