# Correction: The Dynamics of Democracy, Development and Cultural Values

**DOI:** 10.1371/journal.pone.0118878

**Published:** 2015-03-02

**Authors:** The PLOS ONE Staff

There are errors in Equations 9, 10, and 11 in the body of the manuscript and in sections S7, S8, S10 and S12 in S1 File of the published article. Please view the correct equations and correct File S1 below.

$$\frac{dF}{dt}=0.0088\frac{E}{F}$$ (9)

$$\frac{dD}{dt}=0.16+0.041FG-0.048\frac{D}{G}$$ (10)

$$\frac{dE}{dt}=0.028D\left(L-0.585\right)$$ (11)

## Supporting Information

S1 File Supporting Information document with all Supporting Information figures and additional descriptions, explanations and analyses. (PDF)
