# Supplementary material for: The Dynamics of Democracy, Development and Cultural Values
Source: PLoS One. 2014 Jun 6;9(6):e97856. doi: 10.1371/journal.pone.0097856 (PMC4048187; doi:10.1371/journal.pone.0097856)
Supplement: File S1 — Supporting Information document with all Supporting Information figures and additional descriptions, explanations and analyses. (PDF) [file pone.0097856.s001.pdf]

Supporting Information: The dynamics of democracy, development and cultural values

Viktoria Spaiser[1,*], Shyam Ranganathan[2], Richard P. Mann[1,2], David J.T. Sumpter[1,2]
1 Institute for Futures Studies, Stockholm, Sweden
2 Department of Mathematics, Uppsala University, Uppsala, Sweden
∗ **E-mail: Corresponding viktoria.spaiser@iffs.se**

S1. Emancipative vs. Self-expressive values as democracy predictors

We re-run our analysis on human-rights democracy as a function of the Human Development Index (HDI) and cultural values with self-expressive values indicator replacing the emancipative values indicator (for details on emancipative values and self-expressive values indicator see supplementary material *S12*). Our intention was to test, whether the finding that HDI has a stronger effect on human-rights democracy than emancipative values – something that rather deviates from the Human Development Sequence assumptions [1] – is an emancipative values index bias.

The analysis with self-expressive values (S) produced almost the same results. The two-term model (equation 1 in the main text), where HDI (H) has a non-linear positive effect on human-rights democratic change (D), i.e.

$$\frac{dD}{dt} = 0.0709H^2 - 0.0658D$$

remains the overall best model for human-rights democratic change according to the Bayes factor (see Fig. S1). This result shows that HDI is a stronger predictor of human-rights democracy than self-expressive values. If we insist on including it in a model, self-expressive values do however have some positive effect on human-rights democracy too. For example, the comparatively good five-term model with democracy, HDI and self-expressive values as predictors in Fig. S1 is

$$\frac{dD}{dt} = 1.6D + 0.0112\frac{S}{H} + 0.837HD - 0.659\frac{D}{H} - 0.166D^2 \quad (1)$$

However, this model does not result in a better fit overall. The results reported for emancipative values in relation to human-rights democracy are therefore robust to the use of a different, but comparable value indicator, namely self-expressive values.

S2. Model for HDI (Human Development Index)

We run analysis for yearly changes in the Human Development Index (HDI) as a function of HDI itself, human-rights democracy and emancipative values. The results suggest that HDI (H) is neither predicted by human-rights democracy nor by emancipative values. The best way to describe yearly changes in the human development index (dH) according to the Bayes factor (see Fig. S2) is a constant term (equation 3 in the main text):

$$\frac{dH}{dt} = 0.0045$$

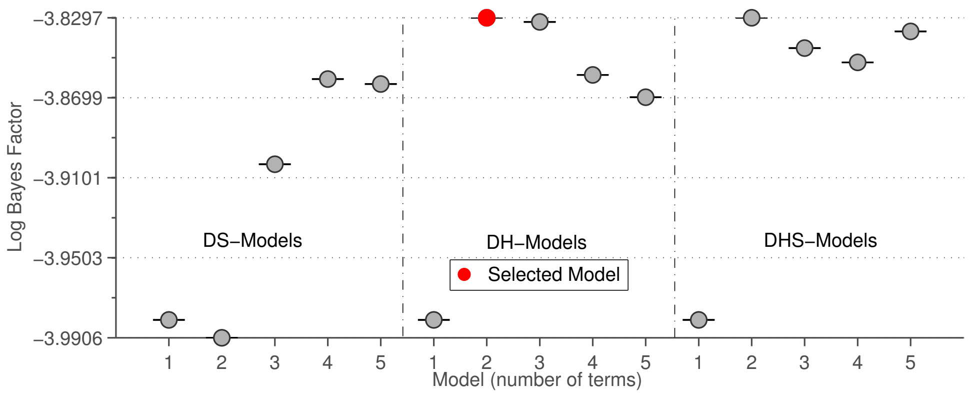

Figure S1. Bayes Factors for models, with human-rights democracy as a function of HDI, self-expressive values and human-rights democracy itself. DS-Models are $f(D, S)$ models with self-expressive values (S) and democracy (D) as predictors for human-rights democracy, DH-Models are models of the form $f(D, H)$ with HDI (H) as predictor and DHS-Models are models including all three indicators, D, H, S. The two-term DH-model (DH(2)) and the two-term DHS-model (DHS(2)) are equivalent and correspond to model 1 in the main text. The five-term DHS-model (DHS(5)) refers to equation 1 in *S1*.

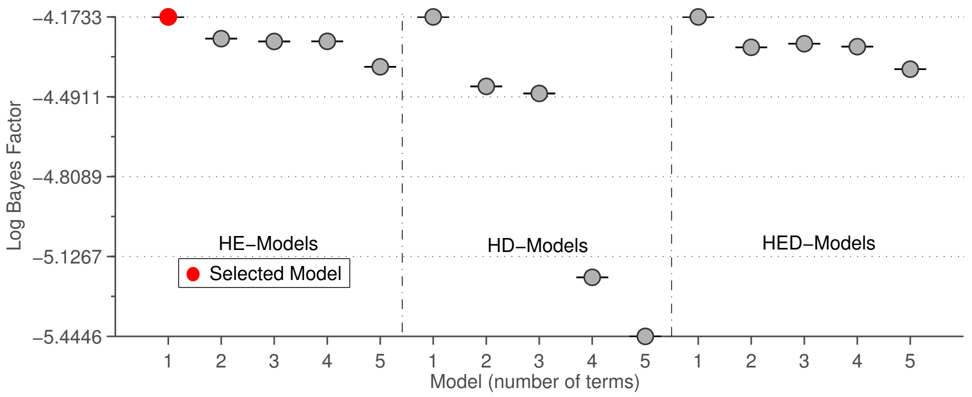

Figure S2. Bayes Factor for models predicting HDI (H), with human-rights democracy (D) and emancipative values (E) as predictors in two-variable models and three-variable model setting. The HE-Models represent models for changes in H as a function of H itself and E, the HD-Models describe changes in H as a function of H itself and D and the HED-Models model changes in H as a function of E and D.The overall best model (Selected Model) refers to the one-term model (HE(1)) with a positive constant term (equation 3 in the main text). The model equals the one-term HD-model (HD(1)) and the one-term HED-model (HED(1)).

S3. Model for Emancipative Values with HDI and Democracy as predictors

We modelled yearly changes in emancipative values (E) as a function of the Human Development Index (HDI) (H) and human-rights democracy (D) in two-variables and three-variables model settings. Baysian

model fitting (see Fig. S3) resulted in the selection of a mathematical model that predicts an increase in a population's emancipative values adoption in proportion to the product of HDI and human-rights democracy (see equation 4 in the main text), i.e.

$$\frac{dE}{dt} = 0.0062HD$$

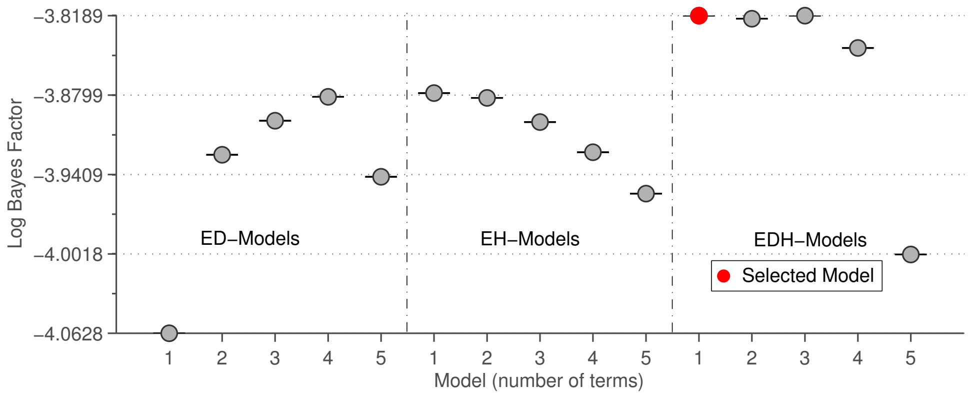

Figure S3. Bayes Factor for models predicting emancipative values (E), with human-rights democracy (D) and HDI (H) as predictors in two-variable modes and three-variable models setting. The ED-Models displays models for changes in E as a function D and E itself, the EH-Models refer to models with H as predictor and EDH-Models represent models for changes in E as a function of H and D and E itself. The overall best model (Selected Model) refers to model 4 in the main text. The comparably good three-term EDH-model (EDH(3)) suggests that human-rights democracy is the primary driver for emancipation and adds some growth-limiting terms, i.e. $\frac{dE}{dt} = 0.118D - 0.0945\frac{ED}{H} - 0.0336\frac{DH}{E}$

S4. Effective Democracy

We repeated our analysis of the relation between democracy, economic development and cultural values, replacing the human-rights democracy index with the effective democracy index (for details on the effective democracy index see *S12* and [2, 3]). Modeling and Bayesian model fitting with the effective democracy measure produces different results. Unlike human-rights democracy effective democracy is not a good predictor for changes in emancipative values. The best model for emancipative values(E) as function of emancipative values themselves and effective democracy (D) according to the Bayes factor (see Fig. S4a) is the following differential equation:

$$\frac{dE}{dt} = 0.0078 + 0.0017E^2 - 0.0047D - \frac{0.0003}{E^2} \quad (2)$$

In this equation effective democracy has merely the function to limit the growth of emancipative values and growth of emancipative values is triggered by emancipative values themselves and by a constant. However, even this "best-fit" model is a rather bad model comparing to models with HDI (H) as sole predictor for changes in emancipative values (E) (see Selected Model in Fig. S4):

$$\frac{dE}{dt} = 0.0136H^2 \quad (3)$$

In this model emancipative values are predicted to grow with HDI and the growth is stronger and faster the higher HDI is.

This finding leads to the conclusion, that effective democracy has no or only a negligible effect on emancipative values. Thus, the crucial component of human-rights democracy for emancipative values to spread in a population appears to be the human rights. The human-rights democracy effects on emancipative values are not time-dependent (at least since 1981). Even when running the analysis with human-rights democracy measure starting only in 1996, that is, taking only the time-period into consideration, that is also covered by the effective democracy measure (1996–2006), we still get the same results with emancipative values being strongly predicted by human-rights democracy in interaction with HDI (see equation 4 in the main text).

Other findings on the other hand are only marginally affected by using effective democracy instead of human-rights democracy. For HDI our original model (equation 3 in the main text) is confirmed as best-fit model (see Fig. S4):

$$\frac{dH}{dt} = 0.0053$$

even though the constant is higher when taking into consideration the period 1996–2006 only.

Also consistent with our previous results, effective democracy itself is best predicted either by HDI solely or by a combination of HDI and emancipative values, while emancipative values on their own are a rather bad predictor for changes in effective democracy. The two best-fit models for changes in effective democracy have similarly high Bayes factors (see. Fig. S4):

$$\frac{dD}{dt} = 0.003H \tag{4}$$

$$\frac{dD}{dt} = 0.068H^2 + 0.123ED + 0.036\frac{HD}{E} - 0.182HD - 0.0218 \tag{5}$$

The first model, which has a slightly lower Bayes factor than the second model, suggests that democracy is growing linearly with HDI. This results differs somewhat from our model for changes in human-rights democracy as function of human-rights democracy and HDI (see equation 1) in terms of linearity of the effect. Probably both, the time interval of 1996–2002 – a period after the democratic transitions that occurred in many countries in the late 1980s and early 1990s – and the different measurement of democracy, have contributed to this different result. However, what is important here, is that comparing HDI and emancipative values as sole predictors, HDI outperforms emancipative values in predicting changes in effective democracy and this is consistent with our main findings.

The slightly better second model draws a more complex picture. In equation 5 HDI has a quadratic positive effect on changes in effective democracy and also a positive interaction effect with effective democracy itself. Emancipative value on the other hand have a positive effect on growing effective democratisation in interaction with the existing level of effective democracy. The other effects are merely growth-balancing effects. This equation may suggest that emancipative values play a slightly bigger role in predicting effective democracy than in predicting human-rights democracy but it is still rather HDI that primarily predicts the rate of changes in effective democracy.

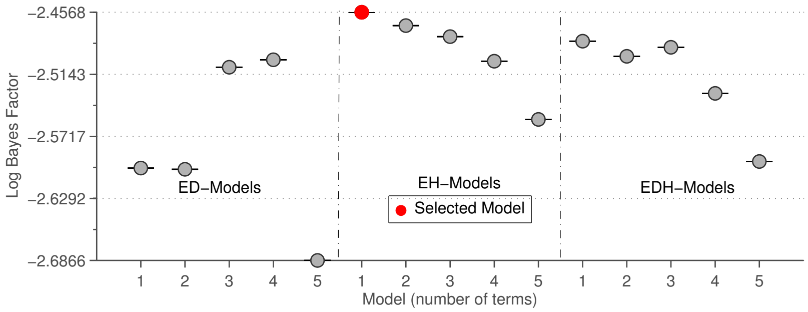

(a)

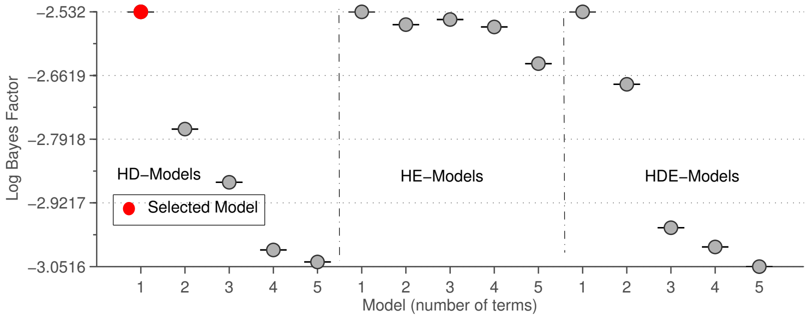

(b)

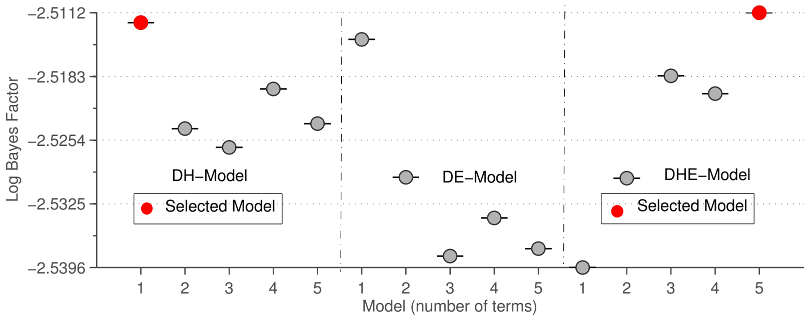

(c)

Figure S4. **(a)** Bayes Factor for models predicting emancipative values (E), with effective democracy (D) and HDI (H) as predictors in a two and three-variable model setting. ED-Models are models for changes in E as a function of D and E itself, EH-Models are models for changes in E as a function of H and E itself and EDH-Models display models for changes in in E as a function of D, H and E itself. The overall best model (Selected Model) represents the model in equation 3. Equation 2 appears in the four-term ED-Model (ED(4)) in this Figure. **(b)** Bayes Factor for models predicting HDI (H), with effective democracy (D) and emancipative values (E) as predictors in two-variable and three-variable model settings, comparing HD-Models of changes in H as a function of H and D with HE-Models of changes in H as a function of H and E, which HDE-Models of changes in H as a function of H, D and E. The Selected Model in (a) represents the overall best-fit model (equation 3 in the main text). **(c)** Bayes Factor for models predicting effective democracy (D), with HDI (H) and emancipative values (E) as predictors in two-variable and three-variable model settings, comparing DH-Models of changes in D as a function of H and D itself, with DE-Models of changes in D as a function of D and E, with DHE-Models of changes in D as a function of D, H and E. The two Selected Models in (b) represents the two best-fit models, given in equation 4 and equation 5.

S5. Education, Life Expectancy and GNI per capita as Democracy Predictors

We computed and fitted models for human-rights democracy (D) as a function of one of the the three components of the Human Development Index (HDI): education (I), life expectancy (L) and GNI per capita (G). From the Bayes factors (see Fig. S5) the three best models predicting human-rights democracy, one model per HDI component, were

$$\frac{dD}{dt} = 0.0448DI + 0.0936D^2 + \frac{0.001}{I} - 0.0407I^2 \tag{6}$$

$$\frac{dD}{dt} = 0.0142 + 0.7234LD + 0.1539\frac{D}{L} - 0.691D - 0.0315L^2 \tag{7}$$

$$\frac{dD}{dt} = 0.074G + 0.3153D - 0.211\frac{D}{G} - 0.29D^2 - 0.026G^2 \tag{8}$$

Education and life expectancy have positive effects on human-rights democracy in interaction with human-rights democracy as predictor, while GNI per capita displays an independent positive effect on human-rights democracy. In all equations a certain level of human-rights democracy itself contributes positively to further democratisation. Finally, all equations show some form of growth limiting terms.

Comparing the predictive power of the three components (pair comparisons: comparing DI- and DL-, as well as DL- and DG-and finally DI- and DG-Models), both, education and GNI per capita turned out to be comparatively good predictors for human-rights democratisation according to the Bayes factor (see Fig. S5). In the second step, we combined the two best predictors, education and GNI per capita in a three-variable model setting (DIG-Models) and found that a model which combines both predictors outperforms those models with education or GNI per capita as sole predictors. Therefore, the best overall model (Selected Model in Fig. S5(c)) predicting human-rights democratisation includes the effects of education and GNI per capita and is displayed in equation 5 in the main text, i.e.

$$\frac{dD}{dt} = 0.11DIG + 0.0248G^2 - 0.119D^2$$

S6. Models for Education, Life Expectancy and GNI per capita

We computed models for all three HDI components with human-rights democracy and emancipative values as predictors in two-variable model settings, that is changes in each component were modelled as (1) a function of the component itself and human-rights democracy or (2) as a function of the component itself and emancipative values.

We found that emancipative values (E) are a good predictor for life expectancy (L) growth, with the best model according to the Bayes factor (see Fig. S6) being the model displayed in equation 6 in the main text, i.e.

$$\frac{dL}{dt} = 0.0283E + \frac{0.0035}{L} - 0.0251\frac{E}{L}$$

In this model emancipative values have a positive linear effect on growing life expectancy. Life expectancy is also increasing by a constant term; this growth is however slowed down by high life expectancy in a society. The overall rising of life expectancy is also limited by a negative term.

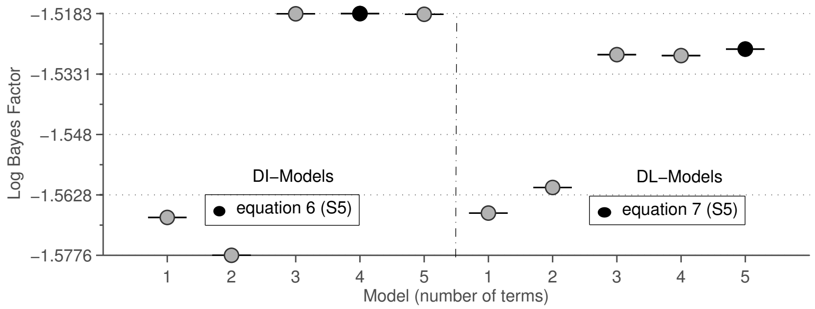

(a)

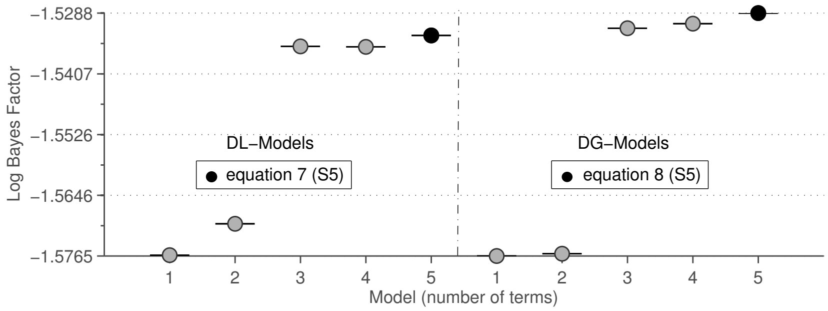

(b)

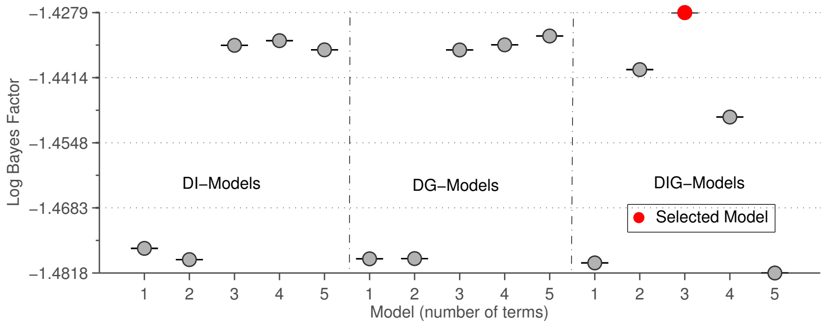

(c)

Figure S5. Bayes Factor for models predicting human-rights democracy (D), with UN education index (I), life expectancy (L) and GNI per capita (G) as predictors in two-variable model settings **(a)** comparing DI-Models of changes in D as a function of I and D itself, with DL-Models of changes in D as a function of L and D; **(b)** comparing DL-Models of changes in D as a function of L and D with DG-Models of changes in D as a function of G and D. The models marked black in a) and b), comparing the two best models of each setting, represent equations 6, 7 and 8 in *S5*. Figure **(c)** finally gives the Bayes factor for the two variable DI- and DG-Models and the three variable DIG-Models predicting changes in D as a function of I, G and D. The Selected Model in (c) represents the overall best model (equation 5 in the main text).

Emancipative values (E) are also a good predictor for GNI per capita (G) even though their impact is rather a negative one. The best model for changes in GNI per capita as function of emancipative values is according to the Bayes factor (see Fig. S6) a model shown in equation 7 in the main text, i.e.

$$\frac{dG}{dt} = 0.0024\frac{G}{E}$$

The model shows that while GNI per capita itself contributes to a further growth of GNI per capita, the growth is slowed down by emancipative values: the higher the level of emancipative values in a society the stronger the slowing down effect on economic growth.

Emancipative values are a rather bad predictor for education (I), with the best model according to the Bayes factor (see Fig. S6) being one where education grows at a constant rate (see equation 8 in the main text):

$$\frac{dI}{dt} = 0.0073$$

The same model is suggested to be the best model for changes in education as a function of human-rights democracy and education itself. Therefore, human-rights democracy is equally a bad predictor for growing education levels, possibly because the education level grows considerably before a society becomes democratic.

Human-rights democracy is also a bad predictor for life expectancy and GNI per capita. The models for changes in life expectancy and for changes in GNI per capita with democracy as predictor, that show high Bayes factors (see Fig.S6) display no term with human-rights democracy (D). Life expectancy (L) is predicted to grow by a constant rate and GNI per capita (G) is predicted to grow as a function of GNI per capita itself:

$$\frac{dL}{dt} = 0.0024 \tag{9}$$

$$\frac{dG}{dt} = 0.0052G \tag{10}$$

S7. Models for female education

We repeated our analysis that involved the UN education index as an indicator with female education. We computed models for changes in female education as a function of female education itself and emancipative values as well as models with female education as a function of female education itself and human-rights democracy. Unlike the UN education index, female education (F) is positively predicted by emancipative values (E). The best model for changes in female education according to the Bayes factor (see Fig. S7) is the model shown in equation 9 in the main text, i.e.

$$\frac{dF}{dt} = 0.0069\frac{E}{F}$$

This model describes the positive linear effect of emancipative values on female education. Growing levels of female education is only slowed down by high levels of female education itself.

Human-rights democracy is a bad predictor for female education, similarly to the UN education index. The model for female education as a function of female education itself and human-rights democracy, that

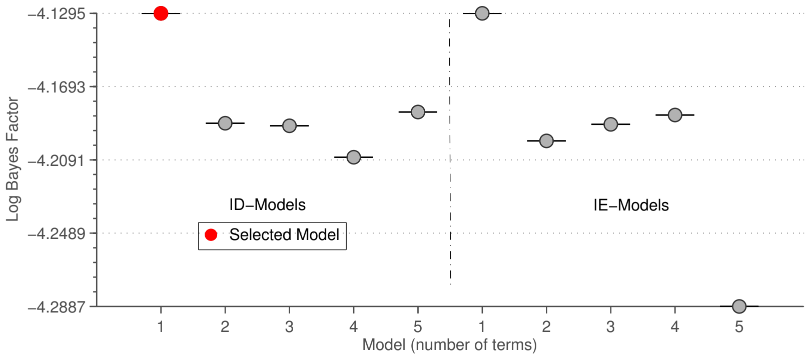

(a)

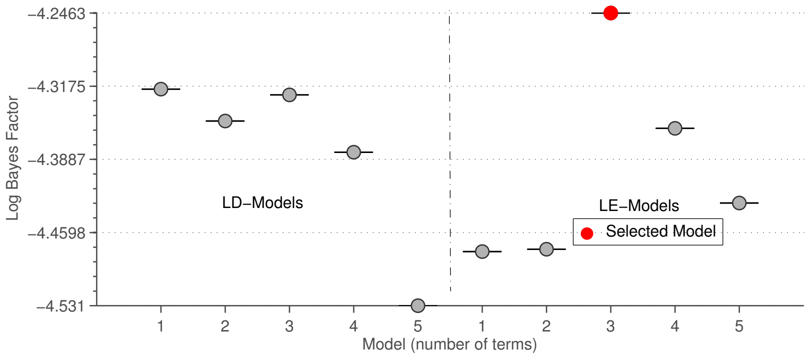

(b)

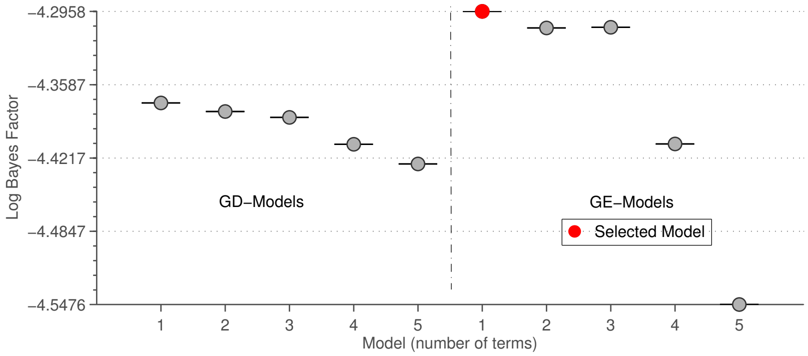

(c)

Figure S6. Bayes Factor for models predicting (a) education (I) with either human-rights democracy (ID-Models) in the function $f(I, D)$ or emancipative values (IE-Models) in the function $f(I, E)$, (b) life expectancy (L) with either human-rights democracy (LD-Models) or emancipative values (LE-Models) as predictors in $f(L, D)$ and $f(L, E)$ and (c) GNI per capita (G) with either human-rights democracy (GD-Models) or emancipative values (GE-Models) as predictors in $f(G, D)$ and $f(G, E)$ in respective two-variable settings. **(a)**The best model (Selected Model) (see equation 8 in the main text), is the same for both model-setting (ID- and IE-Models). **(b)** Equation 6 from the main text is the best model (Selected Model) for changes in life expectancy with emancipative values as predictor. The LD-model with the highest Bayes Factor represents equation 9. **(c)** Equation 7 from the main text is the best model (Selected Model). The GD-model with the highest Bayes Factor represents equation 10.

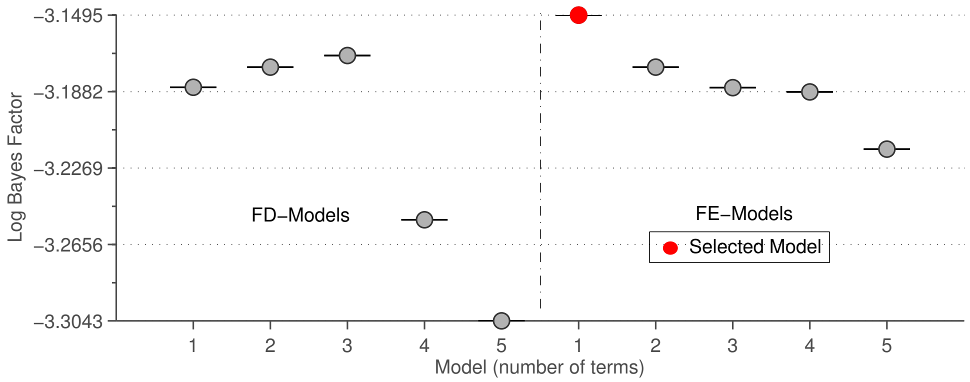

Figure S7. Bayes Factor for models predicting female education. FD-Models display models for changes in F as a function of D and F itself and FE-Models show models for changes in F as a function of E and F itself. The best model (Selected Model) represents the equation 9 in the main text. The model in equation 11 is represented by the the three-term FD-Model (3) in the figure.

is suggested by the Bayesian model fitting, is a model where female education (F) grows as a function of female education itself, while democracy (D) appears only in the growth limiting term, i.e.

$$\frac{dF}{dt} = 0.0013 + 0.0198F - 0.034D^2 \quad (11)$$

The negative non-linear effect of human-rights democracy on female education in this model should be interpreted in terms of a limiting factor only. This is most likely because female education rates are growing considerably before human-rights democracy is build up, that is human-rights democracy cannot predict female education growth. Comparing this model however with models for female education as a function of female education itself and emancipative values shows however, that this model is generally bad. Therefore the conclusion to draw is, that human-rights democracy has no considerable effect on female education.

S8. Female education as democracy predictor

We modelled human-rights democracy as a function of human-rights democracy itself and female education. The Bayes factors (see Fig. S8) suggests a model for human-rights democratic changes (D) with female education (F) having a linear positive effect on human-rights democracy. The equation contains also a growth limiting effect.

$$\frac{dD}{dt} = 0.0488F - 0.0358F^2 \quad (12)$$

Figure S8 shows that the predictive power of the two education indexes, UN education index and female education is quite comparable. It shows that equivalently to the UN education index, female education is a better predictor for human-rights democracy than life expectancy, GNI per capita and GDP per capita. Similar to our analysis with the UN education index, we also combined the two best

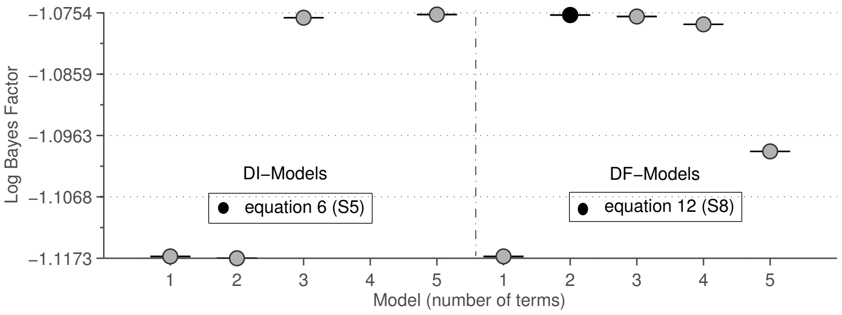

(a)

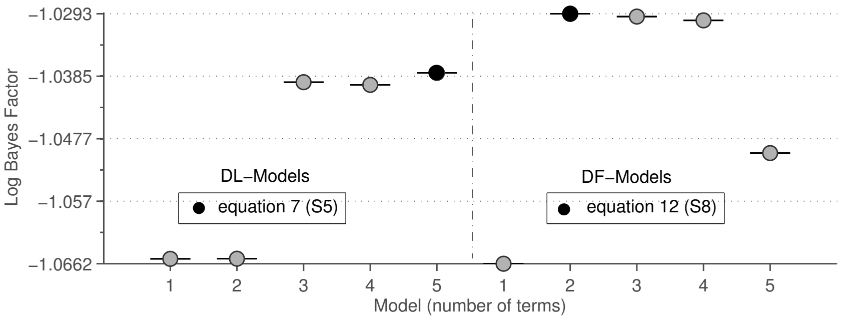

(b)

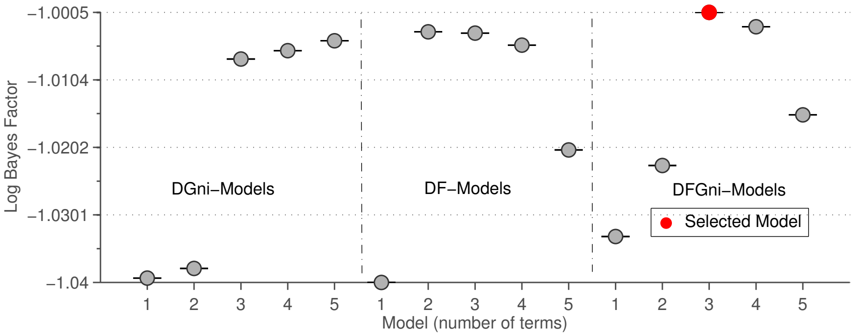

(c)

Figure S8. Bayes Factor in pair comparisons for models predicting human-rights democracy (D), with (a) general education index (I), (b) life expectancy (L) and (c) GNI per capita (G) vs. respectively female education (F) as predictors in two-variable models setting (a, b, c) and a three-variable model setting (c). **(a)** DI-Models represent models for changes in D as a function of I and D itself, DF-Models show models for changes in D as a function of F and D itself. Models marked black, comparing the two best models of each setting, represent equation 6 in *S5* and equation 12 in this section. **(b)** DL-Models display models for changes in D as a function of L and D itself and DF-Models as in (a). Models marked black represent equation 7 in *S5* and equation 12 in this section. **(c)** DGni-Models are models for changes in D as a function of G and D itself, DF-Models as in (a) and DFG-Models finally models for changes in D as a function of G, F and D itself. The best model (Selected Model) in (c) finally represents equation 10 in the main text.

predictors at this analysis step, female education and GNI per capita and tested if a model with both predictors outperforms models with female education (F) and GNI per capita (G) as sole predictors. As Figure S8 shows, this is the case. According to the Bayes factor, the best model for human-rights democratic change (D) taking is the model displayed in equation 10 in the main text, i.e.

$$\frac{dD}{dt} = 0.11DGF + 0.0296G^2 - 0.118D^2$$

This model replicates exactly the model with the UN education index and GNI per capita as human-rights democracy predictors and shows that female education and general education measures are interchangeable when modeling human-rights democratic change.

S9. Education, Life Expectancy and Wealth as Emancipative Values Predictors

To better understand HDI's effect on emancipative values, we computed models for emancipative values (E) as a function of one of the three HDI components: education (I), life expectancy (L) or GNI per capita (G). The Bayesian model fitting procedure (see Fig. S9) resulted in the following three best models for emancipative values, one model per HDI component:

$$\frac{dE}{dt} = 0.05 - \frac{0.0038}{I} - 0.0287\frac{E}{I} - 0.0142\frac{I}{E} \quad (13)$$

$$\frac{dE}{dt} = 0.1343LE + \frac{0.0223}{L} - 0.1287E - \frac{0.0045}{LE} \quad (14)$$

$$\frac{dE}{dt} = +0.1287 + 0.1413EG - 0.159\frac{E}{G} - \frac{0.0139}{E} - 0.2459E^2 \quad (15)$$

Life expectancy and GNI per capita have a positive impact on the growth of emancipative values in a population in interaction with emancipative values themselves, while the positive effect of education appears to be rather weak, mostly counteracting a decline of emancipative values. All equations display some sort of growth limiting terms. Of the three HDI components, life expectancy is the best predictor for emancipative values (see Fig. S9).

S10. Female education as emancipative values predictor

Equivalently to the UN education index, we also modelled emancipative values as a function of emancipative values themselves and female education and found – consistent with our finding on UN education index — that female education has generally a rather weak effect on emancipative values. According to the best-fit models (see Fig. S10) female education (F) seems primarily to stabilise the level of emancipative values (E) in a society, as high levels of female education slow down and at some point stop negative trends in the development of emancipative values levels.

$$\frac{dE}{dt} = \frac{0.006}{E} - \frac{0.0017}{F^2} - \frac{0.0005}{E^2} \quad (16)$$

Similar effects on emancipative values were displayed by the UN education index. Life education remains the best predictors for emancipative values comparing to female education.

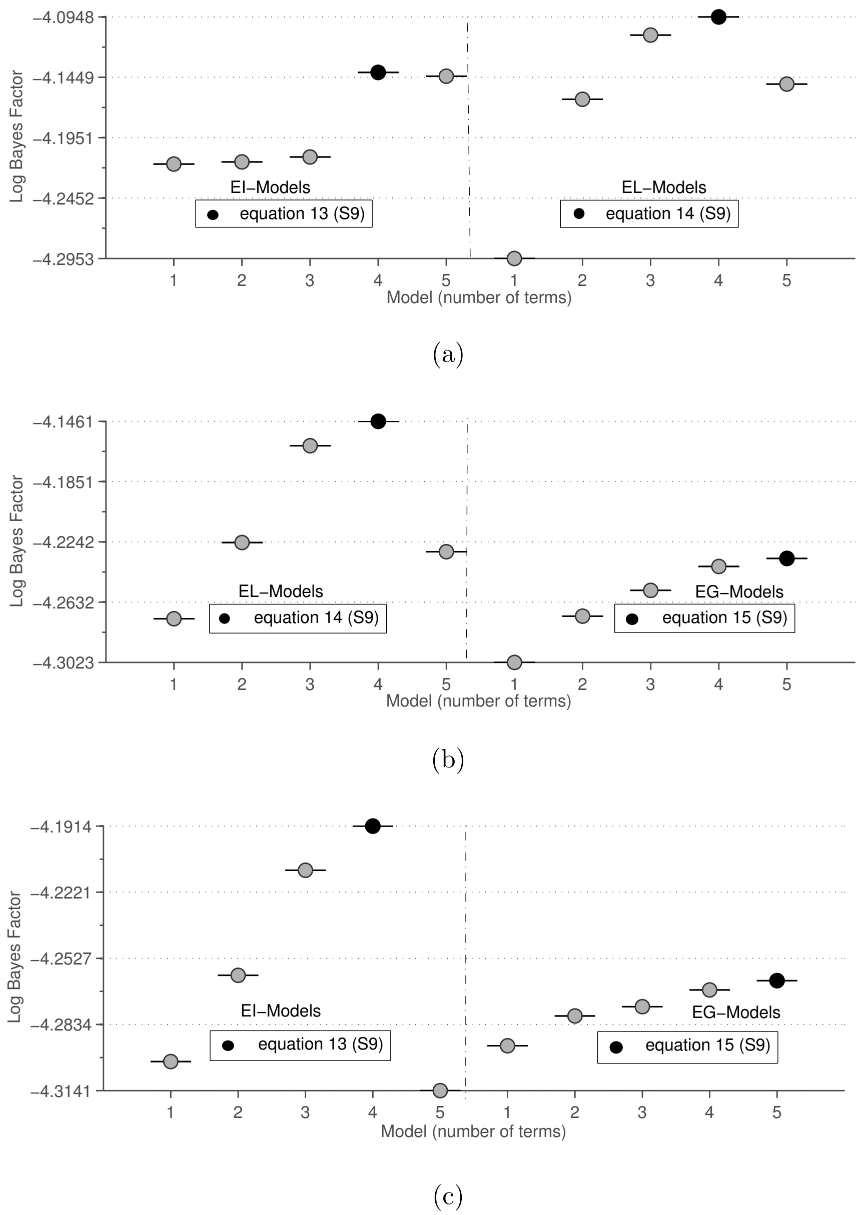

Figure S9. Bayes Factor in pair comparisons for models predicting emancipative values. EI-Models model changes in emancipative values (E) as a function of education (I) and E itself and EL- Models display models for changes in E as a function of life expectancy (L) and E itself. EG-Models show models for changes in E as a function of GNI per capita (G) and E itself. In Figure **(a)** EI- and EL-Model setting are displayed for pair comparison. Models marked black, comparing the two best models of each setting, represent equation 13 and 14. In Figure **(b)** EL- and EG-Model setting are contrasted. Models marked black represent equation 14 and 15. Finally, in Figure **(c)** EI and EG-Model are compared and the black marked models are displayed in equation 13 and 15.

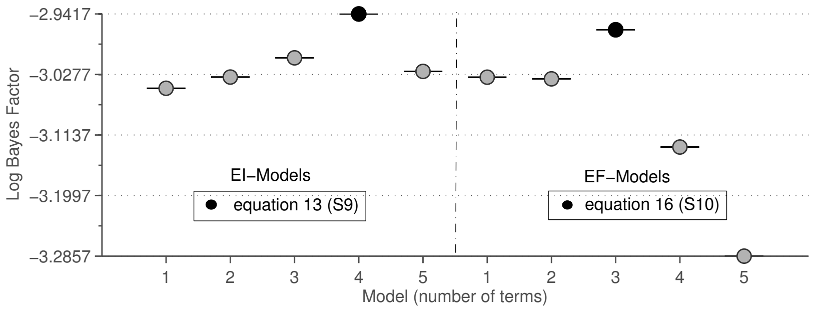

(a)

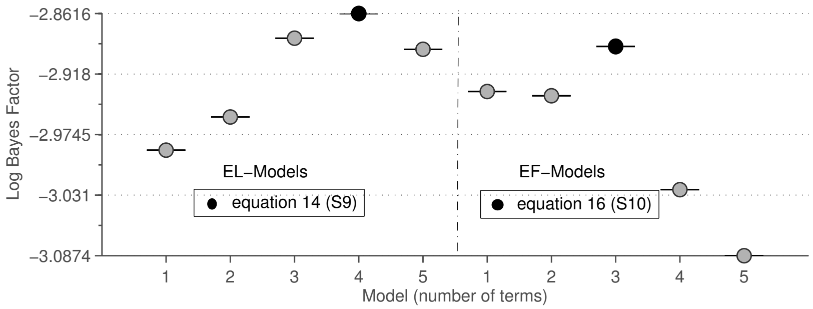

(b)

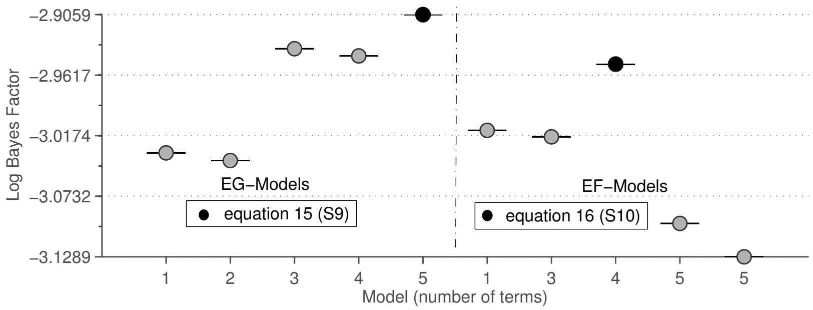

(c)

Figure S10. Bayes Factor in pair comparisons for models predicting emancipative values in two-variable models settings. EI-Models model changes in emancipative values (E) as a function of education (I) and E itself, EF-Models model changes in E as a function of female education (F) and E itself, EL- Models display models for changes in E as a function of life expectancy (L) and E itself, EG-Models show models for changes in E as a function of GNI per capita (G) and E itself. In Figure **(a)** EI-Models are compared to EF-Models. The two black marked models respectively comparing the two best models of each setting represent equation 13 in *S9* and 16 in this section. In Figure **(b)** EL- and EF-Models are compared, with black marked models representing equations 14 in *S9* and 16 in this section. Figure **(c)** contrasts EG- and EF-Models are contrasted, with black marked models representing equations 15 in *S9* and 16 in this section.

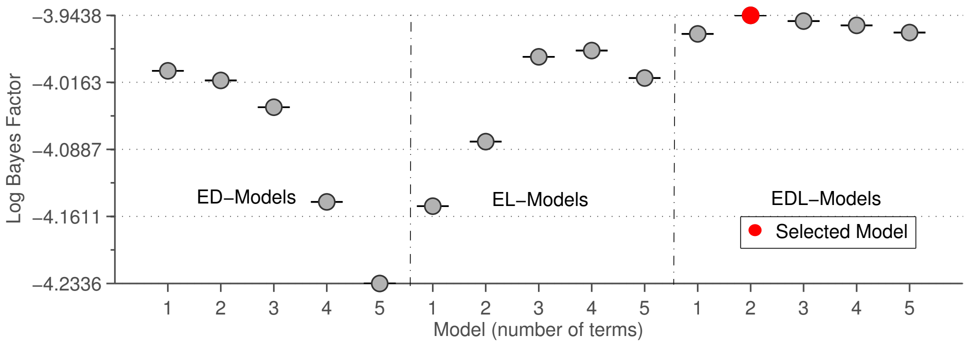

Figure S11. Bayes Factor for models predicting emancipative values (E), with human-rights democracy (D) and life expectancy (L) as predictors. ED-Models are models for changes in E as a function of D and E itself, EL-Models are models for changes in E as a function of L and E itself and EDL-Models display models for changes in in E as a function of D, L and E itself. The Bayes Factor show that the model with both predictors (Selected Model), given in equation 11 in the main text, is better than models with only one predictor, be it D or L.

S11. Life Expectancy and Human-Rights Democracy as Emancipative Values Predictors

At this analysis step we combined the best emancipative values predictor among the HDI components, life expectancy with human-rights democracy, as human-rights democracy is an equal strong predictor for emancipative values rising as HDI (see equation 4 in the main text). We therefore fitted a model for emancipative values (E) as a function of life expectancy (L) and human-rights democracy (D) and compared their fitting to models with life expectancy and human-rights democracy as sole predictors. The best model (see Selected Model in Fig. S11) turned out to be one with both life expectancy and human-rights democracy as emancipative values predictors (see equation 11 in the main text), i.e.

$$\frac{dE}{dt} = 0.028DL - 0.0164D$$

According to this model emancipative values are spreading in a population in proportion to the product of human-rights democracy and life expectancy, limited by a negative term.

S12. Data

S12.1 Socio-economic indicators

We used five variables to measure economic and human development: UN education index (product of mean years of schooling and expected years of schooling), female education (mean years of girls' schooling), life expectancy (in years), log GNI (Gross National Income) per capita and log GDP (Gross Domestic Product) per capita. The UN education index, life expectancy and log GNI are components of the Human Development Index (HDI), which constitutes the geometric mean of the three previously normalised indices [4]. In our main analysis we focus on these three HDI components as well as on HDI itself as indicators. All cross-country time-series socio-economic data were obtained from either UNDP (http://data.un.org) or the World Bank (http://data.worldbank.org). Additional analyses were

carried out on female education, measuring mean years of girls' schooling. The female education index was obtained from the Barro-Lee Educational Attainment Dataset (http://barrolee.com) [5]. Female education was included in the analysis because it is an indicator that education is equally open and accessible to all parts of the population. Moreover female education measures to some degree gender equality.

S12.2. Democracy indicators

There are different ways to measure democracy. In our main analysis we used a human-rights weighted democracy index, which we refer to as *human-rights democracy.* This index is based on Freedom House's civil liberties and political rights scores [6, 7], weighted for the actual human right performance in the respective countries, with two indices from the Cingranelli/Richards Human Rights data project [8, 9]. Freedom House relies mostly on the judgement or regional experts, supplemented by officially documented information to rate societies regarding their civil liberties and political rights. The Cingranelli/Richards human-rights data project (CIRI) indices of personal integrity rights and political empowerments rights on the other hand rely on actual rights violations, documented by the US State Department and Amnesty International. Moreover CIRI uses a standard coding scheme to translate the obtained information into scores that measure to what extent particular types of rights are respected in a society [3].

To build the index, Freedom House political rights and CIRI empowerment rights scores are added to give political participation. Similarly, Freedom House civil liberties and CIRI personal integrity rights are added to give personal autonomy. Political participation and personal autonomy are then multiplied to give the human-rights democracy index. Using a product results in low index scores, making human-rights weighted democracy index a rather rigorous indicator for democracy [3]. To cover the third wave of democratisation in late 1980s and early 1990s, we chose this human-rights weighted democracy index, because it is available for the years 1981 to 2006.

Another democracy measure that is mostly used in the context of Human Development Sequence research [1] is the *effective democracy* index. This index is available only for the years 1996 to 2006. Effective democracy is also based on Freedom Houses's civil liberties and political rights scores, but was weighted by a rule of law index (for more details on the effective democracy index see [10,11]. Specifically, Freedom House civil liberties scores and Freedom House political rights scores were added to give a democracy rights index. Similarly, the World Bank's Rule of Law Index, based on expert judgement and population surveys [12] and World Bank's Control of Corruption Index from the Worldwide Governance Indicators (WGI) dataset were added to obtain an overall rule of law index. The effective democracy index is then a product on the democracy rights and rule of law indices [11].

S12.3. Cultural values indicators

To measure cultural values we used primarily the World Value Survey *emancipative values* index, but also the related *self-expressive values* index. The World Value Survey is a survey of representative sample of citizens from various nations. The survey is carried out by face-to-face interviews or phone interviews in remote areas. The World Values Survey provides longitudinal survey data on the level of individual respondents that may be aggregated on the national level. In our cross-national study we worked solely with aggregated data (for details on aggregation method see http://www.jdsurvey.net).

Emancipative values are measured on four dimensions: choice, equality, voice and autonomy [13]. On the choice dimension respondents are asked to what extent they tolerate abortion, diversity and homosexuality, by for example asking them: 'Please indicate for each of the following statements whether you think in can be always justified, never be justified, or something in between'. The respondents may

express their justification level for 11 items, among others for homosexuality, divorce and abortion on a scale between 1 and 10 [14]. At the equality dimension, three items measure gender equality in the domain of politics, education and jobs. For instance the respondents are asked to what extent they agree with the statement 'A university education is more important for a boy than for a girl', given a five-point Likert scale [14]. To measure the emancipation level on the voice dimension, respondents had to indicate to what extent they demand that people have more say on the local and national level and to what what priority they assign to the freedom of speech. Respondents were asked to choose among four issues what is most and second most important to them. The four issues were: 'Maintaining order in the nation', 'Giving people more say in important government decisions', 'fighting rising prices' and 'protecting freedom of speech' [14]. Finally the fourth dimension autonomy was measured by people's prioritisation of independence and imagination and their rejection of obedience as a desired quality. Surveyed individuals were for instance asked to chose from a list of qualities that children can be encouraged to learn, five. The list contained following items: Independence, hard work, feeling of responsibility, imagination, tolerance and respect for other people, thrift/saving money and things, determination/perseverance, religious faith, unselfishness and obedience [14]. For further details on the emancipative values index see [3].

We run our analysis also with the self-expressive values index [15] to test our results. The self-expressive values index combines a bigger variety of items comparing to emancipative values, even though both value indexes include some core items, namely those on tolerance for homosexuality (and other minority groups), demanding gender equality and prioritisation of independence and imagination while rejecting qualities like obedience. The self-expressive measure does not include items on prioritisation of peoples participation in politics and freedom of speech. Instead self-expressive values include items where respondents were asked if they have had sign a petition or if the respondent has or would joint a boycott. Furthermore, the self-expressive values measure encompasses items on respondent's life satisfaction and overall happiness, respondent's care for environment protection, social trust or respondents' prioritisation of self expression and quality of life over economic and physical security or prioritisation of accomplishment and working with people ones like over good income and job security and finally people's prioritisation of economic liberties over government intervention. For further details on self-expressive values index see [16]. Some scholars expressed critique concerning the technique for measuring values and the validity of the proposed value dimensions [17–19]. We generally acknowledge this critique, but due to a lack of alternative measures we included these measures in our analysis.

Compared to other indicators used in the analysis, the World Values Survey data is limited in several ways. The range of temporal coverage of the countries in World Value Survey varies from a minimum of one wave to a maximum of six waves. We had to exclude all countries where only data from one wave was available, as that kind of data doesn't allow for modelling based on yearly changes in the variables. The 65 countries that remained for analysis however do not represent world's nations correctly. African and Arab countries are clearly underrepresented while European countries are overrepresented. We had data available for many more countries, including African and Arab countries, when it comes to democracy and socio-economic indicators, and we were using this extended data base additionally when modelling the interaction of democracy and socio-economic development. But the overall analysis had to be narrowed to 65 countries to allow for comparisons of the model fits based on Log Likelihood and Bayes factor. Despite these limitations, the World Value Survey is the only longitudinal data set on people's cultural values across a wide variety of societies.

S12.4. Rescaling and Interpolation

We rescaled those indicators that were not already obtained with a range of 0 to 1 for better parametrisation. Log GNI per capita and log GDP per capita which had the same range between 5 and 12 were rescaled by dividing the values in the data by the maximum, that is 12. Life expectancy measure was

rescaled to values between 0 and 1 by diving the data values by 100. Female education was rescaled by dividing the given data for the measure by 13, the maximum of the original female education score range. Effective democracy was equivalently rescaled by a division by 100. Finally, self-expressive values had to be rescaled in two steps. First the self-expressive index values were rescaled by adding 2 to the values, to obtain only positive values ranging from 0 to 4 for the index. In the second step these positively rescaled values were divided by the maximum range scale 4, producing a self-expressive values index scaled between 0 and 1.

The World Value Survey takes place approximately every five years encompassing a time period between 1981 and 2011, however, in our modelling approach we analyse yearly changes in the variables, therefore we had to apply interpolation techniques to get data points between two respective measuring points for each country. We were mainly working with linear interpolation. Specifically, if $x(t)$ was the level of an indicator at year t and $x(t+5)$ is the level 5 years later, then we set

$$x(t+i) = x(t) + \frac{x(t+5) - x(t)}{5} i$$

for the intervening years. Interpolation was in particular necessary for the World Value Survey data, that is for the emancipative values and self-expressive values indicators. Furthermore, data for HDI and UN education index were provided on a yearly basis only from 2000 onwards. Between 1980 and 2000 that data was given only every five years. That made it necessary to apply linear interpolation to these data for the time period of 1980 to 2000 too. The data for all the other indicators was available on a yearly basis.

In preliminary work we additionally used cubic spline interpolation on some sample of the models to test the robustness of our results. We used the Matlab spline function for cubic spline data interpolation (see *http://www.mathworks.se/help/.../spline.html*). For details on the method of cubic spline interpolation see [20]. Cubic spline interpolation did not affect the model results. In particular, the selected models remained the same, although the parameter estimations could change slightly.

References

1. Inglehart R, Welzel C (2005) Modernization, Cultural Change and Democracy: The Human Development Sequence. Cambridge University Press.
2. Welzel C (2003) Effective democracy, mass culture, and the quality of elites: The human development perspective. International Journal of Cultural Studies 43(3-5): 269-298.
3. Welzel C (2013) Freedom Rising. Human Empowerment and the Quest for Emancipation. Cambridge University Press.
4. UNDP (2011) Human development report 2011 - technical notes. Technical report, UN.
5. Barro RJ, Lee JW (2010). A new data set of educational attainment in the world, 1950-2010. National Bureau of Economic Research (NBER) Working Paper 15902.
6. FreedomHouse (2008). Freedom in the world. report. www.freedomhouse.org.
7. FreedomHouse (2010). Freedom in the world. report. www.freedomhouse.org.
8. Cingranelli J, Richards DL (1999) Respect for human rights after the end of the cold war. Journal of Peace Research 36: 511-534.
9. Cingranelli J, Richards DL (2010). CIRI dataset 2008. ciri.binghamton.edu/index.asp.
10. Alexander AC, Welzel C (2011) Measuring effective democracy: The human empowerment approach. Comparative Politics 43: 271-289.
11. Alexander AC, Inglehart R, Welzel C (2012) Measuring effective democracy: A defense. International Political Science Review 33(1): 41-62.
12. Kaufmann D, Kraay A, Mastruzzi M (2006). Governance matters V. World Bank Policy Research Department Working Paper 3630.
13. Welzel C (2006) Individual modernity. In: Dalton RJ, Klingemann HD, editors, The Oxford Handbook of Political Behaviour, Oxford University Press, chapter 10. pp. 185-205.
14. WorldValueSurvey (2006). World Value Survey questionnaire, root version, 2005-2006 wave. http://www.worldvaluessurvey.org.
15. Inglehart R (1997) Modernization and Postmodernization: Cultural, Economic, and Political Change in 43 Societies. Princeton University Press.
16. Inglehart R, Baker WE (2000) Modernization, cultural change, and the persistence of traditional values. American Sociological Review 65: 19-51.
17. Clarke HD, Kornberg A, McIntyre C, Bauer-Kaase P, Kaase M (1999) The effect of economic priorities on the measurement of value change: New experimental evidence. American Political Science Review 93(3): 637-647.
18. Majima S, Savage M (2007) Have there been cultural shifts in Britain?: A critical encounter with ronald inglehart. Cultural Sociology 1(3): 293-315.
19. Klein M, Pötschke M (2000) Gibt es einen Wertewandel hin zum reinen Postmaterialismus? Eine Zeitreihenanalyse der Wertorientierungen der westdeutschen Bevölkerung zwischen 1970 und 1997. Zeitschrift für Soziologie 29(3): 202-216.
20. de Boor C (1978) A Practical Guide to Splines. Springer Verlag.
